# Exploring AlphaFold2′s Performance on Predicting Amino Acid Side-Chain Conformations and Its Utility in Crystal Structure Determination of B318L Protein

**DOI:** 10.3390/ijms24032740

**Published:** 2023-02-01

**Authors:** Haifan Zhao, Heng Zhang, Zhun She, Zengqiang Gao, Qi Wang, Zhi Geng, Yuhui Dong

**Affiliations:** 1 School of Life Sciences, University of Science and Technology of China, Hefei 230027, China; 2 Beijing Synchrotron Radiation Facility, Institute of High Energy Physics, Chinese Academy of Sciences, Beijing 100049, China; 3 University of Chinese Academy of Sciences, Beijing 100049, China

**Keywords:** AlphaFold2, B318L protein, structure determination, molecular docking, side-chain

## Abstract

Recent technological breakthroughs in machine-learning-based AlphaFold2 (AF2) are pushing the prediction accuracy of protein structures to an unprecedented level that is on par with experimental structural quality. Despite its outstanding structural modeling capability, further experimental validations and performance assessments of AF2 predictions are still required, thus necessitating the development of integrative structural biology in synergy with both computational and experimental methods. Focusing on the B318L protein that plays an essential role in the African swine fever virus (ASFV) for viral replication, we experimentally demonstrate the high quality of the AF2 predicted model and its practical utility in crystal structural determination. Structural alignment implies that the AF2 model shares nearly the same atomic arrangement as the B318L crystal structure except for some flexible and disordered regions. More importantly, side-chain-based analysis at the individual residue level reveals that AF2′s performance is likely dependent on the specific amino acid type and that hydrophobic residues tend to be more accurately predicted by AF2 than hydrophilic residues. Quantitative per-residue RMSD comparisons and further molecular replacement trials suggest that AF2 has a large potential to outperform other computational modeling methods in terms of structural determination. Additionally, it is numerically confirmed that the AF2 model is accurate enough so that it may well potentially withstand experimental data quality to a large extent for structural determination. Finally, an overall structural analysis and molecular docking simulation of the B318L protein are performed. Taken together, our study not only provides new insights into AF2′s performance in predicting side-chain conformations but also sheds light upon the significance of AF2 in promoting crystal structural determination, especially when the experimental data quality of the protein crystal is poor.

## 1. Introduction

Traditionally, three-dimensional macromolecular structures are primarily elucidated by X-ray crystallography, cryo-electron microscopy (Cryo-EM), nuclear magnetic resonance (NMR), or a combination of these techniques. Although tremendous efforts have been dedicated to increasingly unraveling protein structures, the procedure of structure determination remains painstaking in large part due to the difficulties of experimentally acquiring stable and pure protein samples on a large scale. As a result, both the released number and deposition speed of novel protein structures in the protein data bank (PDB) are significantly limited [1]. Of particular note, it falls far short of the structural coverage of currently deciphered proteins despite the fact that the Human Genome Project finished plotting the whole gene map of the human proteome nearly two decades ago [2]. To this end, the ability to accurately predict how proteins with complex architectures assemble and fold in three dimensions, based solely on their amino acid sequences, has long been supposed to enable high-throughput structural characterization and thereby hopefully bridge such a dramatic protein sequence-structure gap [3]. However, this possibility has previously been bottlenecked by the relatively low model prediction accuracy or the requirement of highly similar homologous structures. Strikingly, recent advances in artificial intelligence (AI) have aided computational methods in combination with a technique of multiple sequence alignments (MSA) of related proteins to push the prediction accuracy of protein structures to an unprecedented level that is comparable to experimental accuracy [4]. Such machine-learning-based model prediction methods with sufficiently high confidence are powerful enough that they are generally believed to be able to revolutionize structural biology profoundly and open up new avenues for its future development [5].

The outstanding prediction performance of AF2 has been extensively demonstrated in the challenging 14th Critical Assessment of protein Structure Prediction (CASP14) [6,7], which has recently been reinforced with *ColabFold*, a more computationally effective and conveniently accessible platform [8]. The advent of such cutting-edge structural prediction tools has henceforth driven a myriad of interests in further exploring its potential applications in a wide range of fields [9,10,11]. For example, the predicted model that presumably shares high structural similarity to the target protein can be directly used as a search template for molecular replacement (MR) in protein crystallography [12,13,14,15], thus circumventing the stringent requirement for nontrivial chemical modifications or additions to the macromolecule when the target protein has no homologous structures. In the realm of cryo-EM, accurate models can also serve as an important source of structural information for 3D atomic model building [16,17,18] and de novo reconstruction of low-abundance proteins directly from cryo-EM maps [19]. As for NMR, the in silico models can assist with predictions of chemical shifts and building structures amenable to experimentally derived distance and angle restraints [20,21,22]. Recently, impressive progress in the *AlphaFold* protein structure database has been made to massively expand the structural landscapes covering the human proteome and other key organisms (https://alphafold.ebi.ac.uk/, accessed on 24 December 2022), thus providing a far more structural basis for the rational design of mutations and functional analyses of specific proteins [23,24,25]. Collectively, experimental methods in conjunction with accurate computational models can synergize to advance our comprehension of proteins.

Focusing on the field of the ASFV, we attempt to experimentally demonstrate the accuracy and usability of the AF2-predicted model in crystal structural determination using viral prenyltransferases (PTs). Previous studies have confirmed that the B318L protein encoded by the ASFV represents a new member of the viral PT family, according to sequence alignment and a follow-up enzymatic assay [26]. These experiments have shown that the B318L protein catalyzes the condensation of isopentenyl diphosphate (IPP) with allylic diphosphates to produce C20-geranylgeranyl diphosphate (GGPP) and longer chain prenyl diphosphates [26]. It is noteworthy that the B318L protein is identified to be a membrane-anchoring geranyl pyrophosphate synthase (GGPPS) due to its unique N-terminal transmembrane region [27]. The above features raise the hypothesis that the GGPP synthesized by the B318L protein serves as a substrate for the prenylation of cellular or viral proteins and that this post-translational modification is required during virus replication and morphogenesis [28]. Subsequently, it is examined that isopentenyl inhibitors can effectively inhibit ASFV invasion, replication, and release [28]. Taken together, these experiments indicate that B318L is an essential gene for viral replication.

In this study, taking advantage of an accurate model predicted from AF2, the crystal structure of the B318L protein was successfully solved by MR at 3.2 Å. Importantly, based on the new crystal structure, a quantitative performance assessment of AF2 predictions in terms of overall model quality and specific side-chain conformations was analyzed. Moreover, the experimental validation of AF2 predictions in crystal structural determination was performed both quantitatively and comparatively. Finally, the effect of data quality on the crystal structure determination in the context of the AF2 predicted structure was explored and a further structural analysis and molecular docking simulation of the B318L protein were made. We conclude that AF2 predictions can be sufficiently accurate for effective crystal structural determination, whereas some hydrophilic residues, even with high confidence scores, tend to be less accurately predicted on side-chain conformations than hydrophobic residues and therefore should be treated with caution.

## 2. Results

### 2.1. Overall Analysis of AF2 Model against B318L Crystal Structure

**Figure 1:**
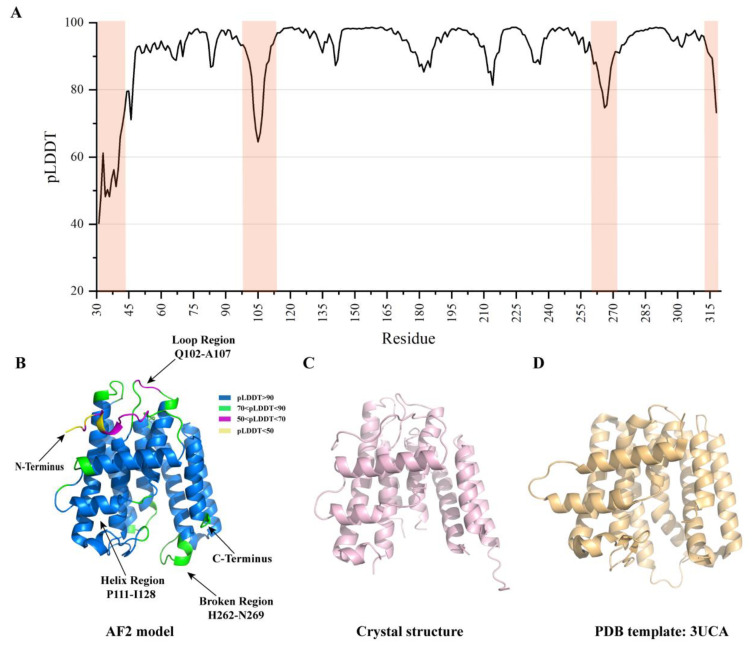
Accuracy analysis of the AF2 predicted B318L structure. (**A**) The predicted pLDDT scores as a function of residue numbers ranging from A31 to T318. The shadowed regions correspond to pLDDT scores less than 80, indicating lower prediction confidence than others. The four regions consist of the N-terminus (A31-N48), the connected linker (Q102-A107), the broken fragment (H262-N269), and the C-terminus (L316-T318). (**B**) Cartoon representation of the structure of B318L as predicted by AF2. The AF2 prediction is color-coded by the pLDDT scores indicating the confidence level of the prediction. Several less well-predicted regions, together with an accurately predicted standard α-helix, are specifically labeled for clarity. (**C**) Cartoon representation of the determined B318L experimental structure. (**D**) Cartoon representation of the closest homolog to the B318L protein with a PDB code of 3UCA.

Despite the great success in determining the crystal structure of B318L with the aid of AF2, a quantitative and in-depth analysis of the accuracy of the AF2 model against the experimental structure is indispensable for a better understanding of its prediction performance. Prior to the availability of experimental structure, the accuracy of the AF2 model could be readily evaluated based on the calculated predicted local distance difference test (pLDDT) scores that correlate well with the confidence level of the model prediction. In general, a pLDDT score greater than 90 is taken as the benchmark for very high accuracy and a pLDDT score greater than 70 corresponds to a generally correct backbone prediction, whereas a pLDDT score lower than 50 indicates unreliable random positions [24]. All five predicted AF2 models have overall pLDDT scores ranging from 91 to 92 and exhibit approximately identical atomic arrangement with a Cα root-mean-square deviation (RMSD) of 0.174 Å on average. The predicted per-residue pLDDT scores of the highest-ranked AF2 model are plotted in Figure 1A, revealing that most residues in the predicted structure have very high confidence scores (pLDDT > 90). In order to structurally visualize the distribution of the pLDDT scores, the predicted B318L structure is color-coded according to the pLDDT scores (Figure 1B). It can be observed that a large portion of the structure can be predicted with sufficiently high accuracy except for certain less conserved regions. The superposition of the predicted model with experimental structure yields an RMSD of 0.574 Å for 213 aligned Cα atoms, demonstrating that they are highly similar (Figure 1C). The homolog with the highest structural similarity to the B318L protein is also compared with our experimental structure (Figure 1D). Although the homologous structure adopts a very similar spatial arrangement of α-helices to the experimental structure, the RMSD between both structures is 2.2 Å for 216 aligned Cα atoms, indicating the remarkable structural difference. These results suggest that the AF2 model holds great promise of being a highly accurate MR search model candidate for crystal structure determination in comparison with homologous structures.

**Figure 2:**
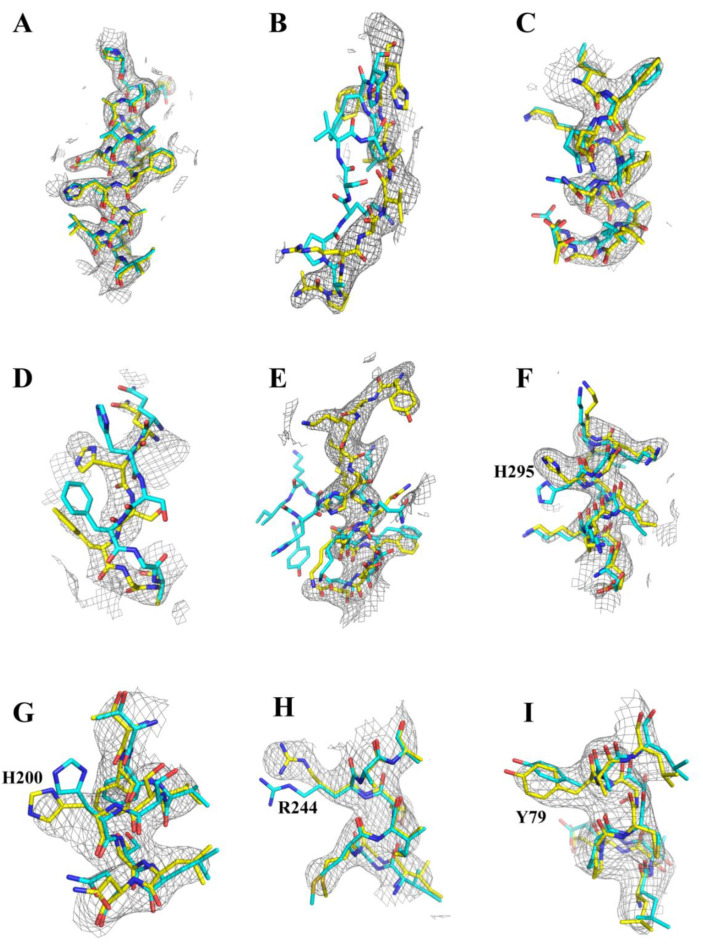
Structural comparison of the unmodified AF2 model against the fully refined B318L crystal structure in some representative regions with the final 2Fo-Fc electron density map overlaid. The fully refined B318L crystal structure and AF2 model are colored yellow and cyan, respectively. The 2Fo-Fc electron density map is contoured at 1.0 σ and represented as a gray mesh. (**A**) A standard α-helix region (P111-I128) showing a nearly excellent agreement between the two models. (**B**) The N-terminus (A31-H39) showing significant backbone deviations. (**C**) The C-terminal region (I307-T318) presenting some minor main-chain displacements. (**D**) The connecting loop region (Q102-A107) exhibiting both main-chain and side-chain discrepancies. (**E**) A highly flexible region (Y270-D282) at the periphery of a piece of the broken structural fragment (H262-N269) displaying completely different conformations. (**F**–**I**) Some selected local regions sharing the same Cα atomic coordinates while adopting remarkably different side-chain orientations with the key residues labeled accordingly. The pLDDT scores for these four residues are 97.3 (H295), 97.9 (H200), 98.2 (R244), and 97.3 (Y79), respectively.

In order to evaluate model accuracy in more detail, some representative fragments of the aligned predicted and experimental structures with the final refined 2Fo-Fc electron density map overlaid are displayed in Figure 2. Overall, most of the electron densities are observed to be well resolved to allow accurate model building, and the AF2 predicted model on the whole adopts very similar or nearly identical backbone and side-chain conformations to the experimental crystal structure (Figure 2A). However, there are still minor structural differences present in some side-chain positions and some flexible loop regions owing to their intrinsically disordered or dynamic features. Such discrepancies usually appear as incorrect side-chain orientations, minor main chain deviations, and unstructured fragments that are commonly found at terminal domains, inter-domain linkers, and peripheral regions devoid of electron density (Figure 2B–I). It is noteworthy that most of these unmatched regions typically correspond to lower pLDDT scores in the AF2 predicted model (Figure 2B–D), which probably originate from crystal disorder, flexible conformations, or even erroneous prediction. These results are overall in good agreement with the above pLDDT analyses of the AF2 predicted model, further confirming the reliability of pLDDT scores for not only measuring model confidence but also identifying potentially disordered protein regions. However, there still exist some exceptions of several amino acid side-chain positions that deviate dramatically from the experimental structure, whereas the corresponding pLDDT scores are unexpectedly high (Figure 2F–I); this is probably due to the origin of the pLDDT score that is calibrated only against the local Cα atomic difference [4]. Therefore, it raises a further challenge for AF2 to accurately measure the prediction confidence of protein side-chains, which will become particularly important since some functions are heavily dependent on the precise arrangements of amino acid side-chains.

### 2.2. Assessing Prediction Accuracy of B318L Protein Side-Chains at the Individual Amino Acid Residue Level

Protein side-chains generally play an important role in specific substrate recognition and ligand binding, thus making them crucial targets for drug discovery. However, accurately predicting protein side-chain conformations remains a challenge and is always hampered by the intrinsic flexibility that multiple possible side-chain orientations for a specific residue can be assumed under different circumstances. In this section, in order to gain a better understanding of AF2′s performance on its ability to model side-chain positions, we evaluated the predicted side-chain confidence by calculating local RMSD values with only aligned non-hydrogen side-chain atoms that are common in both predicted and experimental B318L structures at the individual amino acid residue level.

**Figure 3:**
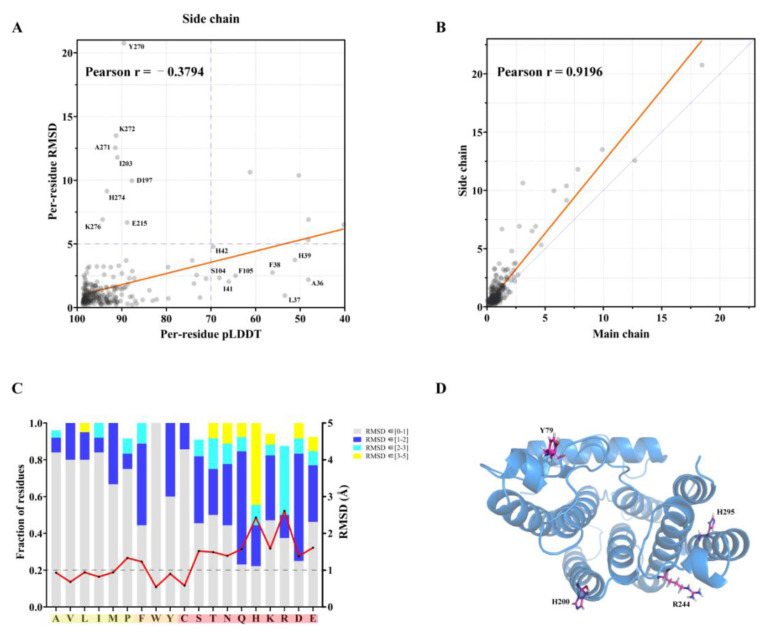
Accuracy analysis of the AF2 predicted B318L protein side-chains. Note that 243 residues extracted from a total of 261 experimentally solved residues are used for side-chain analysis, excluding 18 residues of glycine which comprises only one hydrogen atom in its side-chain. (**A**) A comparison of side-chain RMSD values with per-residue pLDDT scores. Each dot in the scatter plot represents one residue common in both the predicted and experimental B318L structures. Some outliers are labeled for clarity. The red line is the line of best fit. Pearson’s r = −0.3794 and the least-squares linear fit is y=(−0.0879±0.0138)×x+(9.712±1.283). The dotted blue lines indicate a pLDDT score of 70 and RMSD of 5 Å. (**B**) Correlation between side-chain RMSD values and main-chain RMSD values. The red line shows the best fit and the blue line shows x = y for comparison. Pearson’s r = 0.9196 and the least-squares linear fit is y=(1.23±0.034)×x+(0.169±0.071). (**C**) Mean RMSD values for each amino acid type (red line) and the fraction of the residues belonging to the same amino acid type divided into different regions of RMSD values (bar graph). All residues are divided into three groups and colored according to their names, classified into polar (red), nonpolar (yellow), and aromatic amino acids (orange). Note that only residues with side-chain RMSD values less than 5 Å are used for statistical analysis to avoid sufficiently large model bias. (**D**) Examples of representative amino acid residues with apparent disagreement of side-chain positions are plotted on the protein molecule as sticks.

Figure 3A shows the correlation between the calculated RMSD values of side-chain coordinates and the corresponding pLDDT scores for the compared 243 residues in the B318L crystal structure. Overall, most amino acid residues, accounting for nearly 91.4% of all compared residues, are found to fall within the very bottom left part of the plot with pLDDT scores greater than 70 and side-chain RMSD values less than 5 Å. Residues in this region and the upper right region are considered in line with the general hypothesis that accurately predicted residues often have higher pLDDT scores, whereas poorly modeled residues are accompanied by lower confidence. In spite of such consistency, no obvious strong correlation could be made between side-chain RMSD values and pLDDT scores, as evidenced by a relatively low Pearson’s correlation coefficient, possibly due to the dynamic nature of amino acid side-chains. It should also be noticed that several unexpected outliers in Figure 3A show significant discordance between side-chain RMSD values and pLDDT scores. Among them, some exhibit sufficiently high pLDDT scores while corresponding side-chains have large RMSD values, indicating that even residues with very high pLDDT scores cannot be confidently relied upon to reproduce the genuine conformations, particularly when knowledge of amino acid side-chain positions is critical. In contrast, others exhibit low pLDDT scores while their corresponding side-chains are accurately placed, indicating that AF2 occasionally underestimates the prediction confidence for some residues. Next, we analyzed the correlation between the calculated RMSD values of side-chain positions and backbone atoms, which is shown in Figure 3B. In contrast to the relatively low correlation between side-chain RMSD values and pLDDT scores, the side-chain RMSD values correlate well with the backbone RMSD values with a much higher Pearson’s correlation coefficient up to 0.92. It can also be observed in Figure 3B that the least-squared fitted line is significantly steeper than the diagonal along which RMSD values of side-chains are equal to main-chain atoms, indicating that AF2 generally predicts side-chain coordinates with less accuracy than backbone coordinates.

The accuracy of AF2 predicted side-chains may vary remarkably for different kinds of amino acids. To identify whether predicted side-chain accuracy depends on the specific amino acid type, we focused on each type of amino acid and calculated their mean side-chain RMSD values for comparison (Figure 3C). Intriguingly, the effect of amino acid type is glaringly obvious when dividing these amino acids into polar (or hydrophilic) and nonpolar (or hydrophobic) groups. The best predicted amino acids, especially with sub-Angstrom accuracy, tend to be tryptophan, valine, isoleucine, alanine, leucine, and methionine, which are mostly nonpolar and hydrophobic. The worst described amino acids whose side-chain RMSD values noticeably exceed 1 Å are mostly polar and hydrophilic, such as arginine, histidine, lysine, glutamate, glutamine, serine, threonine, aspartate, and asparagine. Among them, positively charged amino acid residues composed of arginine, histidine, and lysine are the most difficult to predict. Unlike the above hydrophilic residues, the polar cysteine can be predicted with sufficiently high accuracy, likely because of its ability to form an intramolecular disulfide bond that can stabilize its conformation. These results are also in good accord with the distribution of residues belonging to the same type of amino acid divided into various RMSD regions (Figure 3C). Figure 3D shows that typical poorly predicted amino acid residues are located on the surface of the protein molecule with their side-chains protruding into the solvent region. The above observation can be explained by the fact that protein folding prefers to bury hydrophobic residues inside the molecule while simultaneously exposing hydrophilic residues on the surface, thus making hydrophobic residues more stable than hydrophilic residues. As a result, buried residues can generally be predicted more accurately by AF2 than surface-exposed residues, which is also consistent with a recent study assessing AF2 performance based on residue solvent exposure [29].

### 2.3. Comparing AF2 Predicted Structure with Other Computational Models

Before the advent of state-of-the-art machine-learning-based prediction methods, computational biologists had to resort to conventional template-based or ab initio modeling programs for structural prediction and bioinformatics analysis. Therefore, it is meaningful to compare AF2 with other AI-based or conventional computational methods with respect to both model prediction accuracy and crystal structural determination. In this section, another six computational models of the B318L protein were also generated using *OmegaFold* [30], *ESMFold* [31], *RoseTTAfold* [32], *SWISS-MODEL* [33], *Phyre^2^* [34], and *i-TASSER* [35] for comparison.

**Figure 4:**
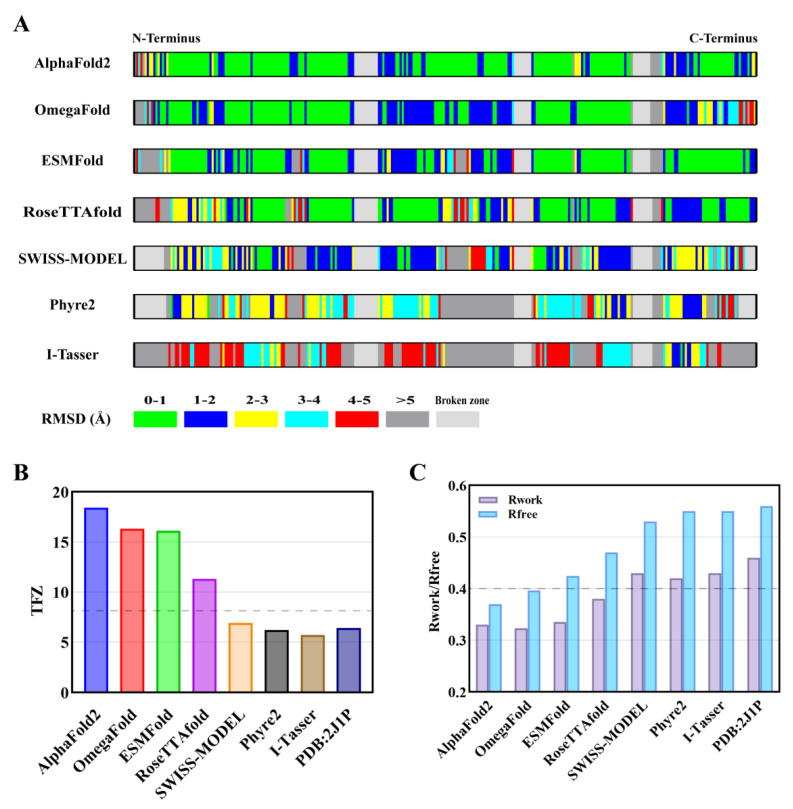
Comparison of different computational models with respect to B318L crystal structure and their applications in crystal structural determination. (**A**) Stripe diagram depicting the calculated per-residue RMSD distribution of each predicted model against the B318L crystal structure after pairwise structural alignment. The series of amino acid residues serve as the x-axis and the N/C termini are annotated at both ends of the stripe. The color codes for different regions of RMSD values are indicated at the bottom left corner. Note that the light gray bar represents broken backbones in the crystal structure that cannot be built due to the lack of observed electron densities or that cannot be predicted in the models. (**B**) A comparison of the best TFZ scores obtained from molecular replacement trials with each model as a search template. The dashed line indicates a TFZ value of 8.0, above which is usually deemed as a correct solution. (**C**) A comparison of paired Rwork and Rfree factors generated by *Phenix.refinement* from each MR solution against experimental data. The dashed line suggests an R factor of 0.4, below which is generally considered a potentially solvable structure.

First, in order to acquire information about the prediction accuracy of each model at the individual residue level, the above six models alongside the AF2 model were aligned against the experimental structure using *Superpose* from the CCP4 interface, which gave rise to a local Cα RMSD value for each pair of aligned residues. The schematic diagram of the calculated full-length RMSD distributions for the seven models with respect to the B318L crystal structure at per-residue level is shown in Figure 4A, suggesting significantly divergent prediction accuracies among various computational methods. Specifically, the AF2 model shares a sufficiently high structural similarity to the experimental structure since the vast majority of its residues fall below the RMSD value of 1 Å. Analogous to the AF2 model, another three AI-based models generated from *OmegaFold*, *ESMFold,* and *RoseTTAfold* also present outstanding performance with a large number of residues predicted with sub-Angstrom accuracy. However, since there are some residues present in these three models exhibiting dramatically higher RMSD values than the AF2 model, other AI-based computational methods are considered slightly inferior to AF2 with respect to model prediction accuracy. On the contrary, very few residues in the other three models produced by conventional methods could coincide well with the experimental structure and there are numerous residues showing pronounced deviations when compared with the AI-based models. Among them, the template-based *SWISS-MODEL* appears to perform best with most RMSD values distributed between 1 Å and 3 Å, possibly due to the existence of numerous homologous templates of the B318L protein, whereas the worst model of the B318L protein comes from *i-TASSER,* with most RMSD values far beyond 4 Å. Taken together, our per-residue RMSD analysis of these seven models implies that the current AI-based computational methods have a large potential to substantially outperform conventional modeling methods in terms of prediction accuracy.

Next, MR trials with each above model as a search template were carried out to further distinguish between their different levels of utility in crystal structural determination. In addition, a homologous structure (PDB code: 2J1P) sharing the highest sequence similarity to the B318L protein with a score of 28% was also used for comparison. After numerous rotation and translation searches for every input model, the translation-function Z-score (TFZ) for each placed model was ultimately given by MR for identifying correct solutions. In general, it is considered that a TFZ higher than 8 reflects a correct solution, and the higher this score is, the more the search model is similar to the target structure. The best TFZs obtained from all MR trials for each search model are shown in Figure 4B. Obviously, the AI-based models generated from AF2, *OmegaFold*, *ESMFold,* and *RoseTTAfold* all have TFZs higher than 8, indicating that these models were correctly positioned and led to successful MR solutions. It is also worth noting that the best TFZ obtained from the AF2 model is appreciably higher than TFZs from other AI-based models, illustrating that the AF2 model performs better than other AI-based models in crystal structural determination. In contrast to the AI-based models, the best TFZs obtained from conventional models are all lower than 8, suggesting that these solutions tend to be incorrect. It is most likely that the large structural deviations between these models and the experimental structure are responsible for their possible failures in crystal structural determination. Irrespective of whether or not the MR solution is correct, an initial refinement of each placed model against experimental data was performed by *Phenix.refine* with the same default parameters to further explore the quality of each MR solution. The resulting pairs of R_work_ and R_free_ factors obtained from the initial refinement for all MR solutions are plotted in Figure 4C. Once again, the solution from the AF2 model is appreciably better than the other solutions, as evidenced by the much lower R_work_ and R_free_ values than those of the best solutions from other models. Of particular note, all AI-based models could produce interpretable and well-connected electron density maps which ultimately contribute to the successful determination of the crystal structure after iterative cycles of refinement (Figure S1A–D). On the other hand, another four solutions generated from conventional models all yielded significantly higher R_work_ and R_free_ values (>40% and >50%, respectively) that are typical of unsuccessful MR trials. As expected, all these solutions make it difficult or even impossible to accurately build crystal structures, owing to numerous chaotic and broken electron densities abundant in both protein backbones and side-chains (Figure S1E–H).

### 2.4. Effect of Data Quality on AF2-Assistant Crystal Structural Determination

The reason for the failure in crystal structural determination using conventional models not only lies in their larger structural deviations but also relates to the poor quality of our experimental data. As can be seen from Table 1, the scaled and merged diffraction data present a very high overall R_merge_ value of 29.7% and its resolution is as low as 3.2 Å. Despite the poor data quality, AF2 still leads to successful structural determination. Nevertheless, it remains unclear how data quality influences crystal structure determination, even with the aid of AF2. In this section, we evaluate the effect of data quality such as data error, data completeness, and data resolution on the AF2-assistant crystal structural determination.

**Figure 5:**
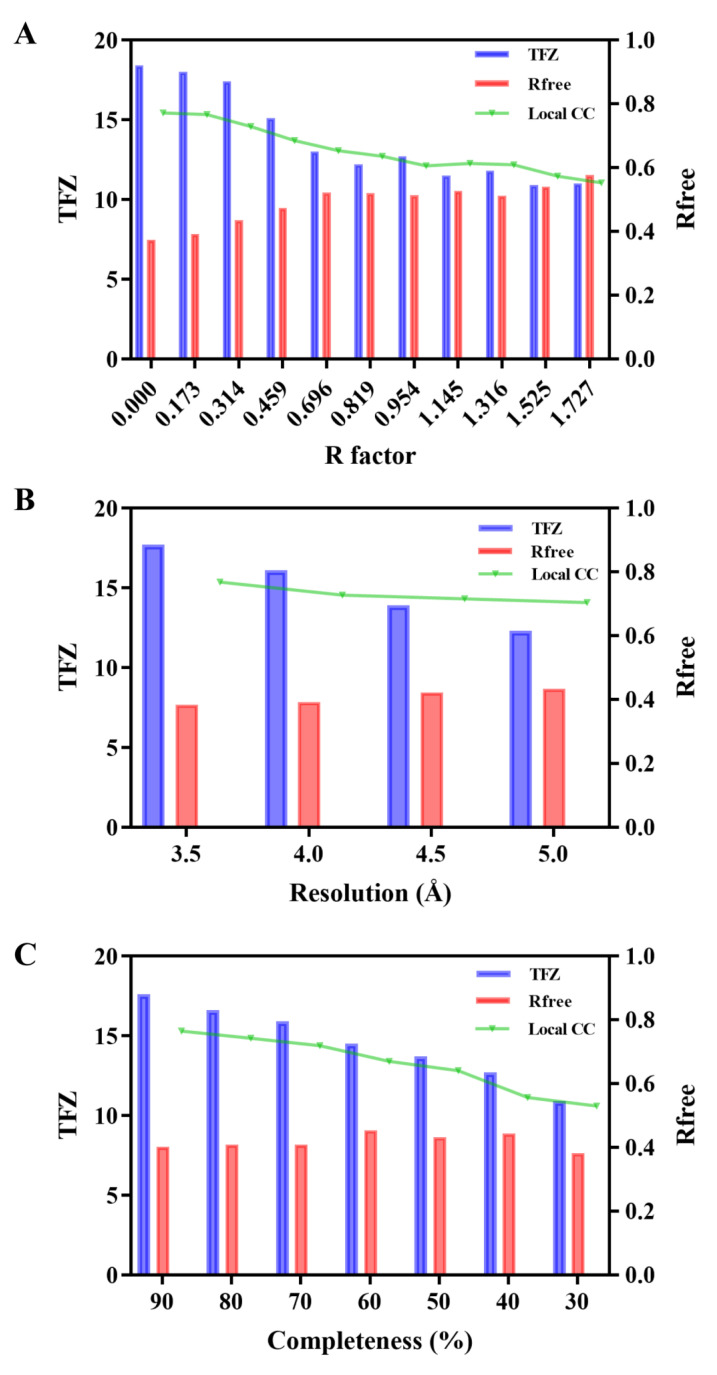
Influence of data quality on crystal structural determination with the aid of the AF2 model. The blue bar represents the best TFZ obtained from each MR trial. The red bar represents the Rfree factor given by *Phenix.refinement*. The green line indicates the local CC between each initially refined electron density map and the final crystal structure. (**A**) Effect of data error. (**B**) Effect of data resolution. (**C**) Effect of data completeness.

First, we numerically corrupted the raw diffraction data with different levels of Gaussian noise. The added errors can be quantified with a classical R factor calculated between the raw data and degraded data. In total, we generated ten sets of degraded data with R factors ranging from 17.3% to 173% for comparison, after which, routine MR with the AF2 model as a search template and initial refinement as above were carried out. In addition to metrics such as TFZ and R_free_, we also adopted the local correlation coefficient (CC) as a quality indicator of structure determination, which is calculated with *phenix.get_cc_mtz_pdb* and used to measure consistency between the electron density map and experimental structure. The effect of data error on structural determination is shown in Figure 5A. It can be observed that all TFZs obtained are higher than 10, indicating the robustness of MR against data error in the presence of an accurate search model. It should also be noticed that TFZ and local CC gradually decrease while R_free_ increases as data error incrementally accumulates, revealing that data accuracy still has a non-negligible impact on structural determination even with a sufficiently accurate AF2 model. Similarly, we also evaluated the effect of data resolution and data completeness on the AF2-assistant crystal structural determination. The results are shown in Figure 5B,C, respectively. Likewise, TFZ progressively decreases with the reduction in both resolution and completeness while always holding its value greater than 10, suggesting correct MR solutions for all these tests. In contrast, both R_free_ and local CC seem to display only small changes regardless of significant resolution alteration, reflecting that AF2-assistant structural determination is least affected by data resolution (Figure 5B). Intriguingly, as shown in Figure 5C, R_free_ is observed to fluctuate only within a small range of around 40%, whereas there is a marked decline in local CC as data completeness decreases. Since local CC directly measures the quality of the electron density map, it is more likely that data completeness has some influence on the AF2-assistant structural determination. Overall, based on the above analyses, we may draw the conclusion that the AF2-assistant structural determination seems not to be susceptible to data error and may even tolerate the data quality of protein crystals to a large extent.

### 2.5. Crystal Structure of B318L Protein and Comparison with Its Homologs

PTs catalyze the consecutive condensation of IPP with allylic diphosphates to produce a variety of prenyl diphosphates with well-defined chain lengths [36]. Generally, PTs are classified into as*cis*- and *trans*-PTs according to the stereochemistry of double bonds from IPP condensation [37]. *Trans*-PTs generally feature an all-helix fold that contains 9-13 α-helices connected with loops. There are two conserved aspartate-rich motifs sitting opposite to each other, forming a large catalytic cavity for allyl substrate binding, termed FARM (the first aspartate-rich motif, DDx2-4D, where x is any amino acid) and SARM (the second aspartate-rich motif, DDxxD). FARM and SARM motifs interact with the substrates via Mg^2+^ [38]. The amino acid sequences of six representative members of the *trans*-PTs family are compared with B318L in Figure S2. These GGPPS share 20% to 25% sequence identities with B318L, indicating a significant evolutionary drift in this family.

**Figure 6:**
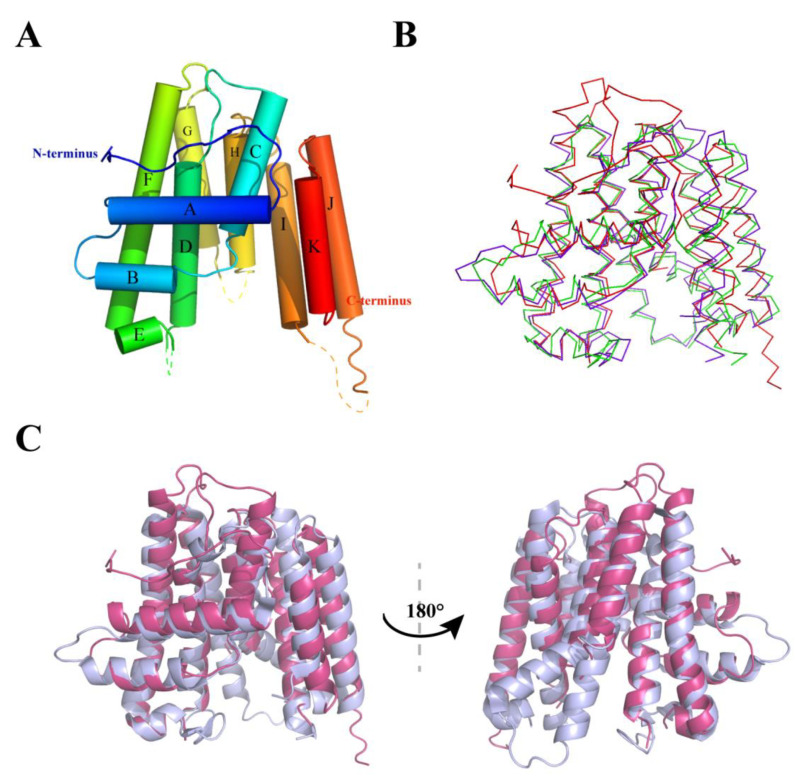
The overall structure of the B318L protein and structural comparison to its homologs. (**A**) Cartoon view showing the overall fold of the ASFV B318L structure. The structural cartoon is colored in rainbow, from blue (N-terminus) to red (C-terminus). (**B**) Superposition of B318L, *Clostridium perfringens* GGPPS, and *Mentha piperita* GGPPS structures. Structures are shown as ribbons and are colored red (B318L), blue (*Clostridium perfringens* GGPPS), and green (*Mentha piperita* GGPPS). (**C**) Superposition of the structures of the ASFV B318L and *Clostridium perfringens* GGPPS in a front view and back view. The structure of B318L and *Clostridium perfringens* GGPPS are shown as a cartoon in red and blue, respectively.

To understand the structural basis of ASFV PTS, we determined the crystal structure of the B318L protein. The full-length B318L gene encodes 318 amino acid residues. For crystallization, the N-terminal putative transmembrane region (residues 1–30) was truncated. Thus, the truncated form of B318L (residues 31–318) was expressed in *E. coli*. As depicted in Figure 6A, the overall structure of B318L adopts a canonical all-helix fold and contains 11 helices (A to K) which can be divided into three layers. The front helical layer is formed by helices A, B, and E. The front layer is orthogonal to the other layers. In the middle layer, there are helices C, D, F, I, and K. The conserved Asp-rich motifs FARM (residues 129–135) and SARM (residues 257–261) are located at helices D and I, respectively. The remaining helices G, H, and J, surrounding the catalytic core, form the back layer. However, there are three loop regions (S133 to K143, Q209 to P214, and H262 to N269) that could not be built due to the lack of interpretable electron density.

We used the DALI server to search for structural homologs of B318L in the Protein Data Bank (PDB) [39]. The best hit is the *C. perfringens* GGPPS (PDB ID: 3UCA) with a Z-score of 22.5 and the second highest score goes to the D chain of the *M. Piperita* GGPPS (PDB ID: 3KRF) with a Z-score of 22.3, both of which correspond to an RMSD of 2.2 Å for 216 equivalent Cα atoms. As shown in Figure 6B, both homologs share nearly the same backbone conformation with the B318L structure, indicating that the ASFV B318L protein is conserved across the *trans*-PTs family. Nevertheless, there are still some conformational variations among the structures. The most significant difference is the various conformations of their N-terminal extensions [40,41], where the N-terminus of B318L starts with a long loop, whereas helices are observed in the N-terminus of other GGPPS (Figure 6B). It is conceivable that the unique N-terminal loop of B318L may extend to its transmembrane region. Another striking difference is that in B318L, the helices G and H in the back layer are significantly shorter than that in the *C. perfringens* GGPPS (Figure 6C).

### 2.6. Molecular Docking with Crystal Structure and AF2 Model for B318L Protein

In order to understand the molecular interactions of GGPP with the B318L protein and further explore the performance of the AF2 model on ligand binding, we performed molecular docking simulations for both the B318L crystal structure and the AF2 model. The calculated binding affinity value for each docking trial is shown in Table 2. The observation that all binding affinity values are lower than a previously reported stringent binding affinity threshold of −7 kcal/mol [42] indicates that all structures can bind tightly with GGPP. Of particular note, the crystal structure of the B318L protein exhibits weaker binding affinity with GGPP when compared with the AF2 model, suggesting somewhat distinct ligand binding capability for the experimental and predicted structures.

**Figure 7:**
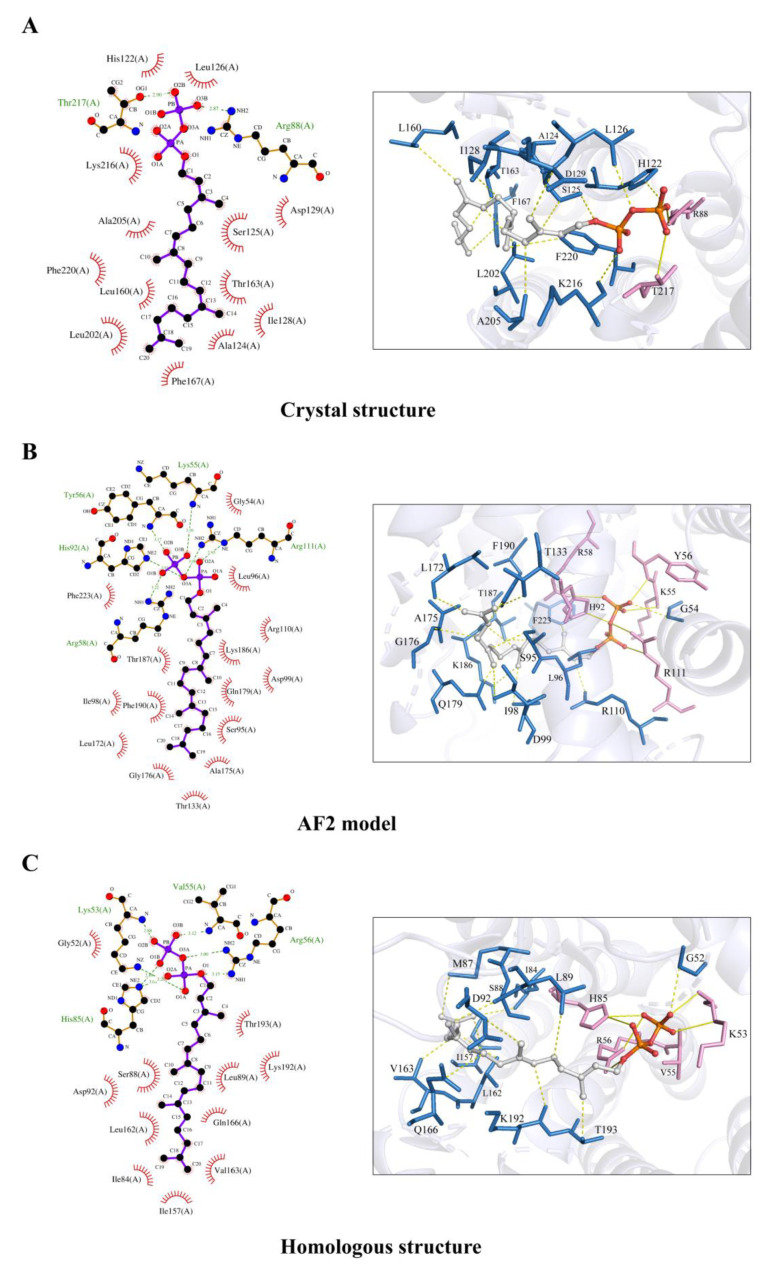
Schematic diagrams of the detailed interactions between the B318L protein and GGPP simulated with molecular docking. (**A**–**C**) Visualization of the detailed molecular interaction of GGPP with the B318L crystal structure (**A**), the AF2 predicted model (**B**), and the 2J1P homologous structure (**C**), respectively. In the left panels, all GGPP molecules and residues for hydrogen-bonding interactions are displayed as stick-ball models and the hydrogen bonds are marked by green dashed lines. All residues for hydrophobic interactions are also labeled and shown as eyebrow symbols. In the right panels, all GGPP molecules are displayed as stick-ball models in gray. Residues for hydrogen-bonding interactions are displayed as stick models in pink and the hydrogen bonds are marked by yellow solid lines. Residues for hydrophobic interactions are displayed as stick models in blue.

Consistent with the above difference in binding affinity values, it is observed that GGPP interacts with the B318L protein at slightly different binding sites for the crystal structure and AF2 model (Figure S3). To identify which binding mode is more likely to occur in the B318L protein, we chose the homologous structure in complex with GGPP (PDB code: 2J1P) as the reference for ligand binding. After overall structural alignment, it is observed that both the predicted binding site and GGPP conformation of the AF2 model coincide better with the homologous structure than that of the crystal structure (Figure S3). To make a more detailed comparison, we further performed molecular interaction analyses for the above two docked structures and the homologous structure using *LigPlot+* (Figure 7A–C). It can be seen that the AF2 model and the homologous structure make more hydrogen-bonding interactions with GGPP than the crystal structure, which is likely responsible for their variations in binding affinity values. Moreover, the AF2 model interacts with the diphosphoric acid of GGPP with highly similar amino acids to the homologous structure, such as histidine, lysine, and arginine, while only arginine is commonly observed to form hydrogen bonds in the crystal structure. Above all, the AF2 model used for molecular docking seems to better describe the GGPP binding mode with the B318L protein than the crystal structure.

**Figure 8:**
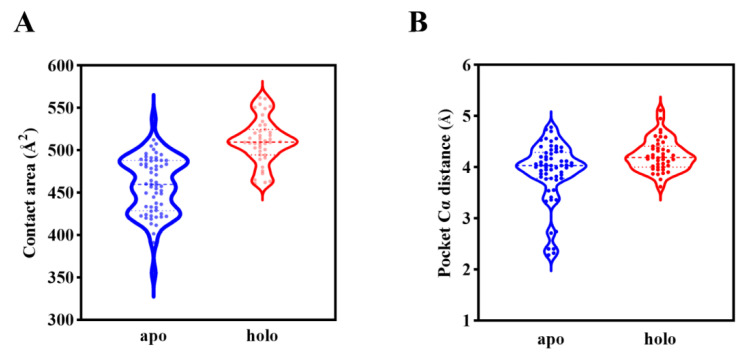
Comparison of the ligand binding capability with respect to both apo and holo templates that are used by AF2 for modeling the B318L protein. (**A**) Distribution of protein-ligand contact areas separated into apo and holo groups. (**B**) Distribution of pocket Cα distances divided into apo and holo groups. The three horizontal dotted lines in each group from up to down indicate the 75th percentile value, median value, and 25th percentile value, respectively. Each dot represents a template structure.

To explore the reason behind such a difference in ligand binding behaviors between the predicted and experimental structures, we carried out an analysis of AF2 templates that were used for modeling the B318L protein. All the templates were divided into ligand-bound and ligand-unbound groups in order to evaluate the possible influence of ligand binding on the performance of AF2 predictions. Templates in the ligand-bound and ligand-unbound states are named holo and apo templates, respectively. All template structures regardless of whether in holo form or apo form were aligned with the AF2 model and further merged with the GGPP that was docked to the AF2 model in order to focus on the same binding pocket for direct comparison. To identify whether the ligand binding capability correlates with different types of template structures, we calculated the protein-ligand contact area for each template (Table S1). Figure 8A shows the distribution of protein-ligand contact areas of AF2 templates that are separated into apo and holo types, indicating the significantly different ligand binding capabilities between apo and holo templates. Notably, it is also observed that the protein-ligand contact areas corresponding to holo templates are remarkably higher than apo templates, suggesting the much stronger ligand binding capability of holo templates. The overall statistical results of the protein-ligand contact areas for the apo and holo templates are displayed in Table 3, together with the AF2 model and experimental structure for reference. Since the experimental structure inherently belongs to the apo form of the B318L protein, its protein-ligand contact area is much closer to the apo templates. Intriguingly, for the AF2 model, its protein-ligand contact area lies between the apo templates and holo templates, with a bias toward holo templates. Since the AF2 model is trained based on structural information from both apo and holo templates, an explanation could be that the AF2 model has learned some knowledge of ligand binding from the holo templates, allowing it to accommodate the ligand in a similar manner that resembles ligand-bound homologs such as 2J1P. In addition, we also compared the pocket Cα distance against the apo and holo templates (Figure 8B), which unfortunately shows a negligible difference between both templates. Therefore, we speculate that the ligand binding pocket may be more correlated with protein side-chain conformations rather than main-chain atoms.

## 3. Discussion

With the advent of the machine-learning-based protein structure prediction software AF2, it is now possible to perform highly accurate structural analyses of functionally important but otherwise structurally unknown proteins [43,44,45]. Additionally, it also holds great promise for facilitating structure-based drug discoveries [42,46,47] and directing site-specific mutagenesis [48,49]. However, further experimental validations of the accuracy of AF2 predictions are still required, thus necessitating the development of integrative structural biology in synergy with both computational and experimental methods. In this study, benefiting from an in silico model predicted by AF2, we successfully determined the crystal structure of B318L at a resolution of 3.2 Å. However, common MR trials using several homologs with the highest similarity scores and other models generated from traditional computational methods have all failed to yield correct solutions, as manifested by the low TFZ and high R factors during structural determination. Though slightly inferior to AF2, other cutting-edge AI-based prediction methods, such as *OmegaFold*, *ESMFold*, and *RoseTTAfold*, have also been found to provide a correct MR solution and eventually lead to successful structural determination. These results are further confirmed by quantitative and in-depth analyses of each model regarding the final crystal structure based on per-residue RMSD comparisons and careful inspections of each resolved electron density map. To this end, it can be anticipated that MR, further armed with highly accurate AI-based predicted models, will undoubtedly dominate the future crystal structural determination of individual proteins and even protein complexes, thereby making traditional derivative-based methods such as anomalous scattering and isomorphous replacement gradually secondary.

It has been extensively demonstrated that the AF2-predicted models present sufficiently high accuracy on average, however, predictions specific to side-chain conformations are always less accurate [50,51,52]. In our study, it is observed that there are some residues showing significant deviations in the predicted side-chain positions whereas their confidence scores given by AF2 are unexpectedly high. That is to say, pLDDT scores alone cannot accurately measure whether some side-chain orientations are correct, which may hinder the analysis of potential active sites for drug design. Therefore, further development of AF2 is needed to improve its prediction accuracy, especially for side-chain conformations, and to propose a more reliable criterion to measure side-chain confidence. In addition, our side-chain analysis at the individual residue level reveals that AF2′s performance is likely dependent on the specific amino acid type, with hydrophilic amino acids predicted less accurately than hydrophobic amino acids. As a result, more attention should be paid to hydrophilic residues when using AF2 models to predict potential binding sites or interpret protein interaction mechanisms. In particular, we also find that positively charged amino acid residues are the most difficult to predict and further speculate that buried residues can potentially be predicted more accurately by AF2 than surface-exposed residues. Therefore, the predicted residues with less accuracy in side-chain positions should be treated with more caution in the further analysis of AF2 models, especially when protein side-chain conformations play an important role.

Despite its spectacular structural modeling performance, AF2 is at the moment hampered by its limited abilities to accurately predict how multiple protein components assemble into functionally important integral machinery and how peptide-protein interactions or site-specific mutations induce conformational changes [53,54,55,56]. For example, it has recently been reported that AF2 shows limitations in predicting the assembly between extracellular domains and transmembrane domains for the family of G protein-coupled receptors which delineates a common paradigm of typical flexible multi-domain structures [51]. Although there have been some studies showing AF2′s potential applications in protein complex predictions [57,58,59,60] and mutational analyses [48,49], more experimental validations are certainly indispensable. Currently, AF2 is still unable to model protein structures in complexes with nucleic acids or reveal structures binding chemical compounds, which is of fundamental importance for identifying protein interaction sites and for future drug discovery [46]. Moreover, AF2 generally provides a single static picture of a protein structure that it thinks is most likely to appear in the PDB, whereas proteins exist in many different conformations, and AF2 currently cannot capture all aspects of a protein’s biological function [61,62]. Taken together, experimental techniques currently remain the only methods capable of tackling biologically important challenges associated with complex formation, ligand binding, and conformational dynamics. Nevertheless, with the ongoing improvement of machine-learning-based model prediction algorithms, it is expected that AI-based techniques will not only make progress toward more accurate and versatile structural predictions but may also promote increased structural diversity in deciphering a protein’s interaction mechanisms with various types of substrates.

## 4. Materials and Methods

### 4.1. In Silico Model Predictions of B318L Protein

A total of seven in silico models of the B318L protein were generated in this study for comparison, including the state-of-the-art machine-learning-based models from AlphaFold2, OmegaFold, ESMFold, and RoseTTAfold, and three conventional models from SWISS-MODEL, Phyre^2^, and i-TASSER. Conventional models were predicted from their available web servers with the query sequence as the only input information. The OmegaFold model was predicted from the BioLib platform, a library of biological data science applications (https://biolib.com/protein-tools/omegafold, accessed on 24 December 2022). The ESMFold model was retrieved from the prediction server provided by the ESM Metagenomics Atlas. The AF2 model was predicted on a local computer equipped with AF2 which was installed as instructed with all databases downloaded and accelerated with two NVIDIA GeForce RTX 3090 GPUs. The input information to AF2 only included the query sequence and the default running parameters were used during prediction. A total of five independent models ranked by pLDDT scores indicating the confidence level of the predictions were produced by AF2 and the model with the highest pLDDT score was selected for further analysis. No additional refinement or truncation was performed on the above in silico models.

### 4.2. Plasmid Construction

The full-length DNA sequence encoding the ASFV B318L (Uniprot accession number Q65164) was purchased from GenScript Corp. (Nanjing, China). The gene lacking 30 N-terminal residues (referred to as B318LΔN30) was cloned into the bacterial expression vector pET28a (Novagen, Madison, WI, USA), fused with a hexa-histidine tag and a SUMO tag at the N-terminus. The sequences of the primers were: 5′- ACAGATTGGTGGATCCGCACCGCGTAGTGTCG-3′ (forward) and 5′-TGGTGGTGGTGCTCGAGTTAGGTCCCCAATGCAACATTTATA-3′ (reverse). The accuracy of the inserts was verified by sequencing. The recombinant vector was then transformed into *Escherichia coli* BL21 (DE3) competent cells (Invitrogen, CA, USA) for protein expression.

### 4.3. Protein Expression, Purification, and Crystallization

The frozen recombinant strains were revived in Lysogeny broth (LB) medium supplemented with 50 μg/mL kanamycin at 37 °C overnight. Every 5 mL revived bacterium suspension was inoculated into 1 L LB medium supplemented with 50 μg/mL kanamycin and grown to an optical density at OD600 of 0.6 to 0.8 at 37 °C. Protein expression was induced by adding isopropyl-β-D-1-thiogalactopranoside (IPTG) at a final concentration of 0.1 mM, and then the induced cultures were grown at 16 °C for an additional 18 to 20 h. The cells were harvested by centrifugation at 5000× *g* for 35 min at 4 °C. The cell pellets were resuspended in Buffer A (20 mM Tris pH 8.0 and 300 mM NaCl) and were further lysed with a low-temperature ultra-high pressure cell disrupter (JNBIO, Guangzhou, China). The lysate was clarified by centrifugation at 10,000× *g* for 60 min at 4 °C. The supernatant was purified by affinity chromatography with nickel-charged IMAC resin (GE Healthcare, Chicago, IL, USA). The fusion protein was eluted from the column using Buffer B (20 mM Tris pH 8.0, 300 mM NaCl, and 300 mM imidazole). The fractions containing the desired fusion proteins were dialyzed against Buffer A at 4 °C overnight, and Ulp1 protease was added to the protein solution during the dialysis process. The mixture was then loaded again onto the Ni-NTA column and the untagged proteins were washed using Buffer A supplemented with 30 mM imidazole. Proteins were further purified by gel filtration chromatography using a Superdex 75 column (GE Healthcare, IL, USA). The column was equilibrated in Buffer C (20 mM Tris pH 8.0 and 100 mM NaCl) using an ÄKTA purifier system (GE Healthcare, USA). The highly purified protein fractions were pooled and concentrated to 12 mg/mL using a membrane concentrator (Millipore, MA, USA).

Initial crystallization screening was performed manually using several commercial crystal screen kits (Hampton Research, Aliso Viejo, CA, USA). The crystallization experiments were conducted at 20 °C, using the sitting-drop vapor diffusion method. The crystals of B318LΔN30 were obtained with precipitant conditions using Crystal Screen 2 NO. 36 (0.1 M HEPES pH 7.5, 4.3 M NaCl).

### 4.4. Experimental Data Collection and Structural Determination

X-ray diffraction data were collected on the beamline BL10U2 at the Shanghai Synchrotron Radiation Facility (SSRF). All crystals were cryoprotected using their reservoir solution supplemented with 20% (vol/vol) glycerol and snap-frozen in liquid nitrogen before data collection. A total of 360 diffraction frames were recorded on a DECTRIS EIGER X 16M pixel array detector. The exposure time and oscillation range per frame were set to 0.1 s and 1°, respectively. Diffraction data were processed using *XDS* and merged and scaled using *XSCALE* [63]. The crystal structure of B318L was determined by molecular replacement using *Phaser* [64] with the highest-ranked AF2 model as the search template. Only one molecule could be positioned in the asymmetric unit according to the Matthews coefficient analysis. The structure was refined via iterative cycles of refinement performed using *Phenix.refine* [65] and manual model rebuilding using *Coot* [66]. Data collection and refinement statistics details are shown in Table 1. The program *PyMOL* (http://www.pymol.org, accessed on 24 December 2022) was used to prepare structural figures.

### 4.5. Structural Alignment and RMSD Calculations

The overall structural alignment between any pair of protein structures was accomplished with the “align” function in *PyMOL*, which yields an overall Cα RMSD value. In order to calculate the main-chain RMSD value for each aligned residue pair, we adopted the *Superpose* software from the CCP4 interface, which outputs a list of per-residue main-chain RMSD values. To calculate the per-residue RMSD value concentrating only on amino acid side-chains, we modified and ran the Python script “pairwise_dist.py” (https://pymolwiki.org/index.php/Pairwise_distances, accessed on 24 December 2022) within the *PyMOL* environment to compare only equivalent non-hydrogen side-chain atoms and output the averaged side-chain RMSD value for each residue pair after overall structural alignment. The atoms used for side-chain RMSD calculation are only present in amino acid side-chains, excluding main-chain C, O, N, and Cα atoms.

### 4.6. Molecular Docking

We used *AutoDock Vina 1.2.0* [67] to dock GGPP against the B318L crystal structure and AF2 predicted model for comparison. The molecular structure of GGPP was extracted from the homologous structure (PDB code: 2J1P) and saved in a single PDB format file. To acquire a more accurate docking result, a proper grid box centered at the potential active site should be specified for docking. Since the B318L protein structure shares some similarities with the PDB structure of 2J1P, we hypothesized that the binding sites of GGPP for both proteins should also be similar. As a result, we defined the coordinates of GGPP in the homologous structure as the center of the cubic box with a grid size of 42 × 42 × 42 Å and further aligned both the B318L crystal structure and AF2 model to the homologous structure. To prepare the protein and ligand for docking, we next used *AutoDock Tools* [68] to convert each PDB file into *AutoDock Vina*’s PDBQT format. For receptor preparation, we deleted all water molecules and added polar hydrogen atoms to the protein structure. The Gasteiger charges model was further used to add partial charges to the receptor. For ligand preparation, we assigned bond torsion as either rotatable or non-rotatable and directly saved it into a PDBQT format file.

Docking was performed with a default exhaustiveness of 32, which specifies the number of runs that start with a random ligand conformation, and a default n_poses of 9, which specifies the final number of ligand poses to report. To demonstrate the validity of our docking simulations, we re-docked GGPP against the PDB structure of 2J1P with GGPP artificially removed. The predicted best pose with the lowest binding affinity of −7.8 kcal/mol was compared with the known structure using *PyMOL* and we observed a good agreement between the predicted and experimental GGPP binding conformations, confirming the feasibility of our molecular docking. To further improve the quality of our docking predictions, we repeated the molecular docking for each case multiple times, aiming to find the best-predicted pose with the lowest binding affinity. Finally, the predicted ligand pose with the lowest binding affinity was selected as the docking model for further analysis. Receptor-ligand interactions were analyzed using LigPlot+ (v2.2) [69].

### 4.7. Analysis of AF2 Templates Used for Modeling B318L Protein

The templates used by AF2 for training the B318L model are listed in Table S1, which are further grouped into ligand-bound structures (in holo form) and ligand-unbound structures (in apo form) for comparison. The probability of each template for AF2 prediction is also shown.

To identify the potential binding pocket in each template, all templates were structurally aligned to the AF2 model which was docked with GGPP as above. For holo templates, the originally bound ligand was removed. After that, each aligned template was merged with the docked GGPP to form a synthetic structure in a complex with the same ligand for further analysis. To quantitatively measure the relationship between each template and docked ligand, the protein-ligand contact area as well as the mean distance between the ten nearest neighbor protein Cα atoms and the docked ligand (pocket Cα distance) were calculated for each template. To calculate the protein-ligand contact area for each synthetic complex structure, the Python script “contact_surface.py” (https://pymolwiki.org/index.php/Contact_Surface, accessed on 24 December 2022) was run within the PyMOL environment. For calculation of the pocket Cα distance, we modified and ran the Python script “distancesRH.py” (https://pymolwiki.org/index.php/DistancesRH, accessed on 24 December 2022) within the PyMOL environment using only protein Cα atoms adjacent to the docked ligand. The resulting protein-ligand contact area and pocket Cα distance for each template are also listed in Table S1.

## 5. Conclusions

In summary, our study reports the crystal structure of the B318L protein which was experimentally determined with the aid of an accurately predicted AF2 model. Under the condition of relatively poor data quality and low sequence similarity, attempts to adopt search models generated from previous modeling methods as well as several homologs with the highest similarity scores have all failed. The quantitative structural comparison demonstrates that the AF2 model is overall in good agreement with the B318L crystal structure except for some intrinsically disordered loops and unstructured regions. Our side-chain-based analysis at the individual amino acid residue level suggests that side-chain RMSD values tend to have a weak correlation with corresponding pLDDT scores and AF2 generally predicts side-chain coordinates with less accuracy than backbone coordinates. More importantly, it is observed that AF2′s performance is presumably dependent on the specific amino acid type and hydrophobic residues are apt to be more accurately predicted than hydrophilic residues. In addition, our molecular docking simulations against both experimental and predicted B318L structures suggest that the AF2 model unexpectedly seems to be more suitable for correctly binding GGPP, in large part, due to its ability to learn structural information from ligand-bound homologs. Looking forward, we envision that AF2 will have a prominent impact on future structural biology and help to accelerate our understanding of life sciences that require structural knowledge.

## Figures and Tables

**Table 1:** Data collection and refinement statistics.

ASFV B318L NΔ30	
Data collection statistics	
Data collection	SSRF BL10U2
Wavelength (Å)	0.9792
Resolution (Å)	48.2–3.2 (3.28–3.20)
Space group	*P*6_5_22
Unit cell parameters	a = 56.13Å, b = 56.13 Å, c = 376.69 Å, α = β = 90°, γ = 120°
No. of unique reflections	6579 (472)
Completeness (%)	99.7 (99.2)
Redundancy	31.7 (28.3)
Mean *I*/σ (*I*)	15.6 (2.2)
Molecules in asymmetric unit	1
R_merge_ (%)	29.7 (251.3)
R_meas_ (%)	30.2 (256.0)
CC_1/2_	0.998 (0.718)
Structure refinement statistics	
R_work_/R_free_ (%)	24.6/28.4
Number of atoms	2058
Protein residues	261
Root-mean-square deviations	
Bond length (Å)	0.008
Bond angles (°)	1.421
Ramachandran plot	
Favored (%)	94.5
Allowed (%)	5.1
Average B-factor (Å) of protein	99.5

**Table 2:** The binding affinity values for three different structures in complex with GGPP.

Protein Structure	Binding Affinity (kcal/mol)
Crystal structure of B318L	−7.2
AF2 model of B318L	−7.8
Homologous structure (2J1P)	−7.8

**Table 3:** Statistics of protein-ligand contact areas for apo and holo templates. The AF2 model and experimental structure are also shown for reference.

	Apo	Holo	AF2 Model	Crystal Structure
Mean value (Å^2^)	458.56	510.84	498.8	462.6
Standard deviation	34.83	26.4	0	0
No. of structures	61	43	1	1

## Data Availability

The structural data of B318L has been deposited in the PDB under accession code 8HDL.

## Supplementary Materials

The following are available online at https://www.mdpi.com/article/10.3390/ijms24032740/s1.
