# Supplementary material for: Exploring AlphaFold2′s Performance on Predicting Amino Acid Side-Chain Conformations and Its Utility in Crystal Structure Determination of B318L Protein
Source: Int J Mol Sci. 2023 Feb 1;24(3):2740. doi: 10.3390/ijms24032740 (PMC9916901; doi:10.3390/ijms24032740)
Supplement: Supplementary file 1 [file ijms-24-02740-s001.zip › ijms-2104186-supplementary.pdf]

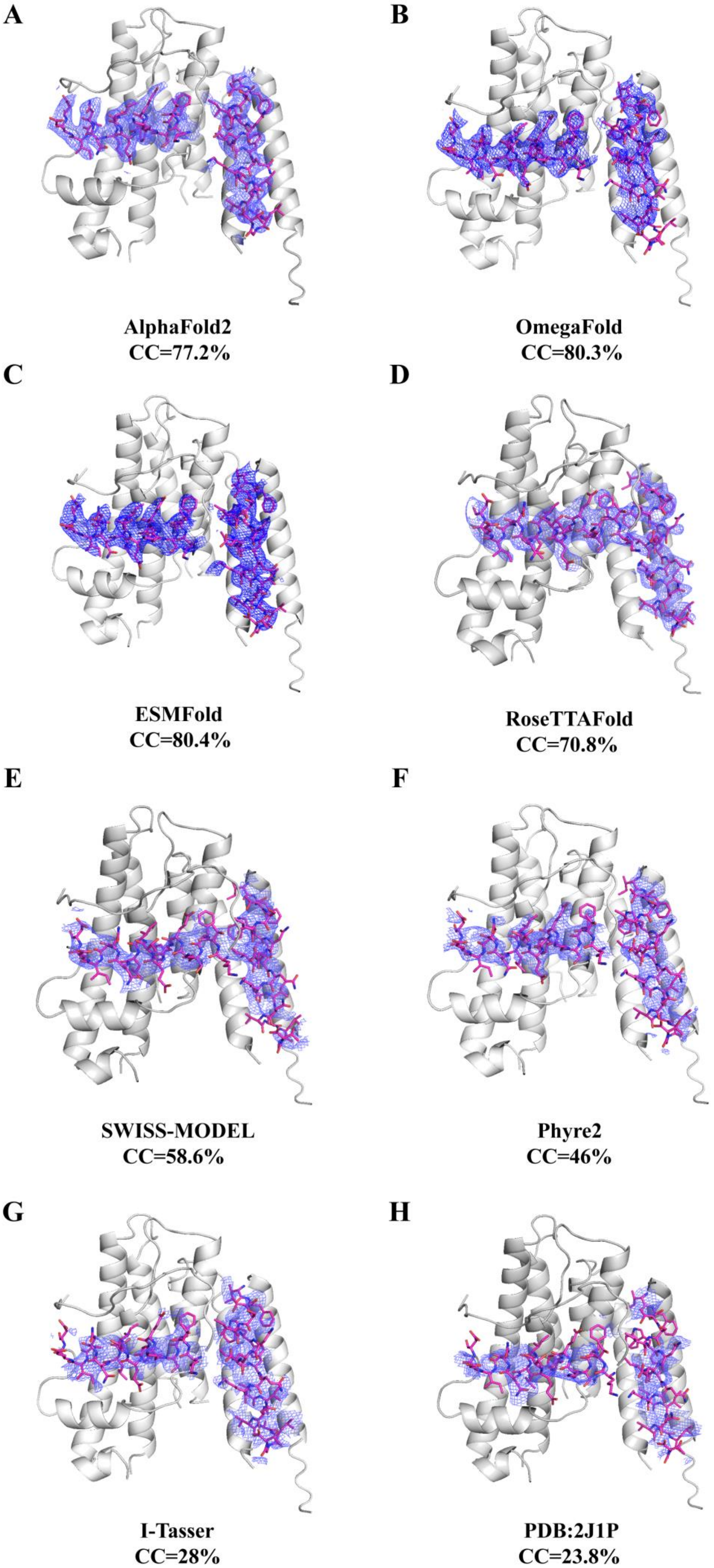
A
B
AlphaFold2
CC=77.2%
OmegaFold
CC=80.3%
C
D
ESMFold
CC=80.4%
RoseTTAFold
CC=70.8%
E
F
SWISS-MODEL
CC=58.6%
Phyre2
CC=46%
G
H
I-Tasser
CC=28%
PDB:2J1P
CC=23.8%

Figure S1. Comparison of different electron density maps displayed at two selected α-helices with the final resolved B318L crystal structure superimposed. Note that each map is obtained from an initial refinement with Phenix.refinement after MR. The 2Fo-Fc electron density map is contoured at $1.0\ \sigma$ and represented as a blue mesh. (A-D) Electron density maps from AF2, OmegaFold, ESMFold and RoseTTAfold showing well-resolved densities in both backbones and side-chains which are in line with the crystal structure. (E-F) Electron density maps from SWISS-MODEL and Phyre2 indicating remarkably deteriorated map quality where only a few side-chains can be visually recognized. (G-H) Electron density maps from i-TASSER and a PDB structure (code: 2J1P) suggesting the worst map quality characterized by significantly broken backbones. The correlation coefficient (CC) between each electron density map and crystal structure is also calculated and labeled under each image for a quantitative comparison.

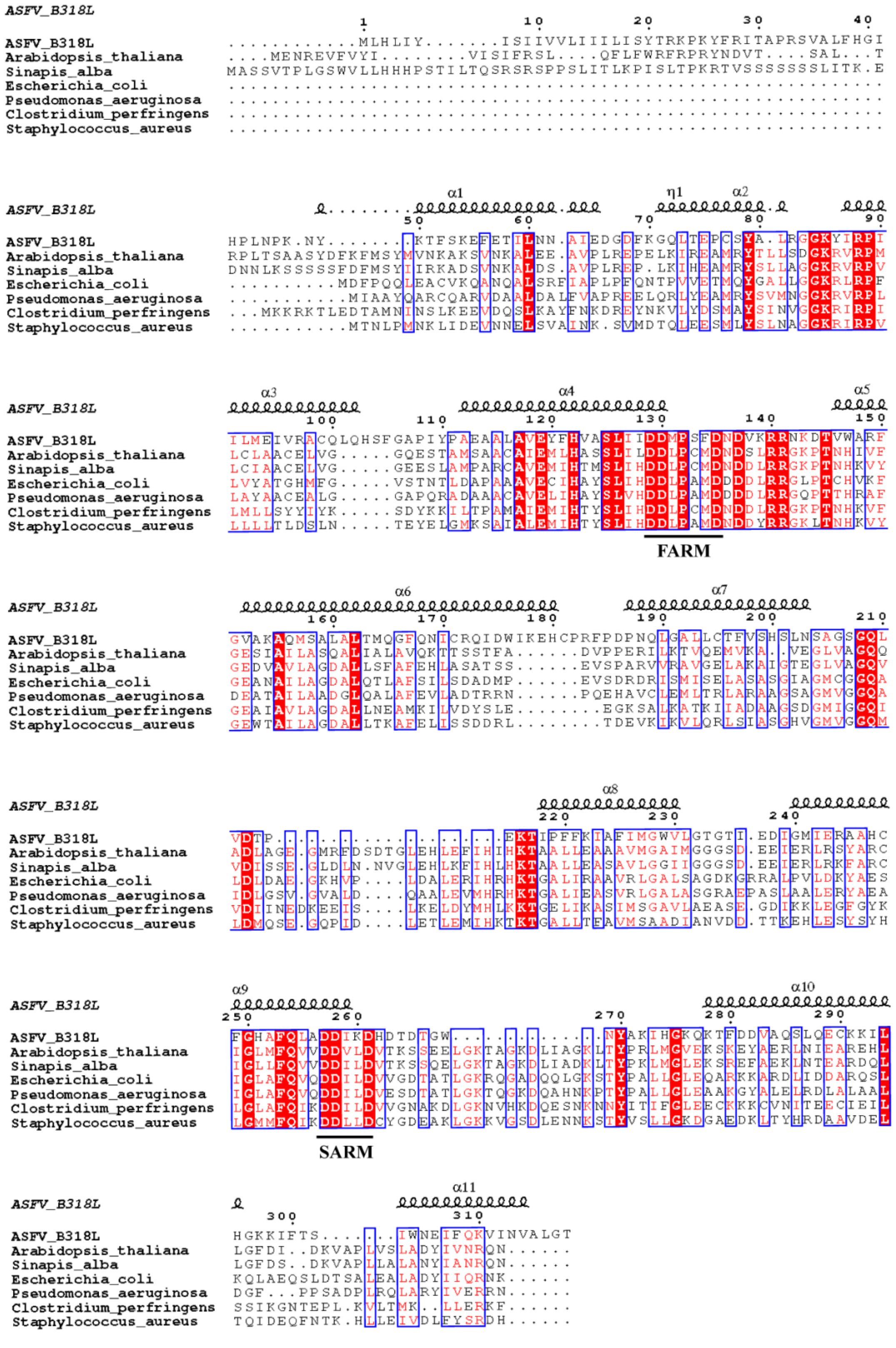

Figure S2. Multiple sequence alignment of B318L protein homologs. Structure-based sequence alignment of B318L with GGPPs from different species performed using Clustal W and ESPript 3. The conserved residues are boxed in blue, and identical conserved residues are highlighted by red letters. The conserved catalytic motif FARM and SARM are highlighted by underlines.

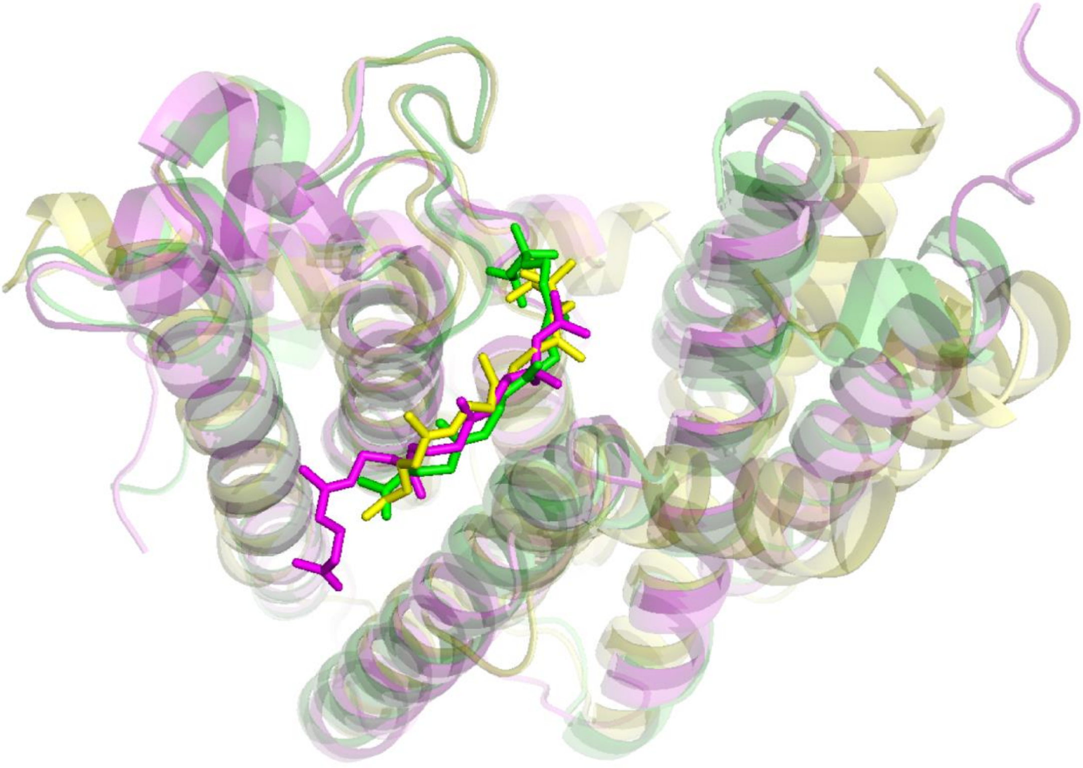

Figure S3. Structural alignment of different structures in complex with GGPP. The crystal structure of B318L docked with GGPP is shown in magenta. The AF2 predicted model of B318L docked with GGPP is shown in green. The homologous structure (PDB code: 2J1P) in complex with GGPP is shown in yellow as the reference.

Table S1. List of AF2 templates together with the calculated pocket Cα-ligand distances and protein-ligand contact areas after placing GGPP into the same position of each template.

PDB ID	Chain ID	Probability	apo/holo	Mean pocket Cα distance (Å)	S.t.d Pocket Cα distance(Å)	Contact area ($Å^2$)
5XN6	D	100	apo	3.55	0.92	433.6
4F62	A	100	apo	3.99	0.45	424
4F62	B	100	apo	3.81	0.47	421.3
2H8O	A	100	apo	3.93	0.54	449.5
3TS7	A	100	apo	3.9	0.57	494.9
3TS7	B	100	apo	3.75	0.76	488.3
5XN5	A	100	apo	4.03	0.39	483.8
4KKM	B	100	apo	4	0.32	436.4
3LVS	A	100	apo	4.03	0.65	481
3M0G	A	100	apo	4.17	0.58	477.4
3KRA	D	100	apo	4.31	0.43	488
3LLW	D	100	apo	4.2	0.63	505.1
1RTR	A	100	apo	4.2	0.65	491.4
3LOM	A	100	apo	4.4	0.56	459.8
3UCA	A	100	apo	4.04	0.61	471.1

5AYP	B	100	apo	4.04	0.4	422.7
3M9U	C	100	apo	4.12	0.62	478.7
3LSN	A	100	apo	4.03	0.39	449.7
3P8L	A	100	apo	3.78	0.63	460.4
3NPK	A	100	apo	4.05	0.53	483.3
4WK5	A	100	apo	4.08	0.56	507.6
3IPI	A	100	apo	4.7	0.67	444.9
3MZV	B	100	apo	4.1	0.54	487.4
3ZOU	B	100	apo	3.95	0.5	452.3
2AZK	B	100	apo	4.74	0.95	432.6
2AZJ	B	100	apo	4.56	1.01	411.5
3LK5	A	100	apo	3.41	0.99	425
3P8R	A	100	apo	4.39	1.13	401.5
3AQB	D	100	apo	4.34	0.6	537.1
3WJK	B	100	apo	4.26	0.52	448.9
1WY0	A	100	apo	4.36	0.42	488.6
3KRA	C	100	apo	3.78	1.14	488
3KRC	C	100	apo	3.82	1.08	473.8
3QKC	A	100	apo	3.36	1.18	420.1
3QKC	B	100	apo	3.36	1.11	421.1
4JXY	A	100	apo	3.85	0.46	428.2
4JYX	A	100	apo	3.87	0.55	464.3
1WL0	A	100	apo	2.32	1.06	422
3LMD	A	99.96	apo	4.12	0.84	456.8
1V4E	A	99.96	apo	2.4	1.15	418.9
2O1O	A	99.96	apo	2.28	0.56	391.2
3TC1	A	99.96	apo	3.95	0.56	355.5
3TC1	B	99.96	apo	2.71	0.85	489.3
1WL2	A	99.96	apo	2.4	1.12	422.5
4LOB	A	99.96	apo	3.33	0.91	433.2
2DH4	A	99.95	apo	4.41	0.55	492.7
5JFQ	B	99.95	apo	3.87	0.92	480.6
5ERN	A	99.95	apo	4.42	0.41	501.2
3MAV	D	99.95	apo	4.3	0.68	437.5
1FPS	A	99.95	apo	4.78	0.72	458.6
6V0K	A	99.95	apo	2.74	0.83	413.3
1WMW	A	99.94	apo	3.77	1.34	497.3
3RMG	A	99.94	apo	4	0.97	429.1
4DHD	A	99.94	apo	3.99	0.53	496.1
2J1O	A	99.94	apo	4.06	0.66	491.2
1YHK	A	99.94	apo	4.56	0.72	481.4
3APZ	B	99.94	apo	3.54	0.78	462.1
3NF2	A	99.94	apo	4.15	0.68	483.5
4KK2	A	99.94	apo	4.51	0.69	457.5

4KK2	B	99.94	apo	4.53	0.72	454.9
5H9D	A	99.93	apo	4.28	0.73	512.3
3Q1O	A	100	holo	3.9	0.89	489.3
2FOR	A	100	holo	4.06	0.74	472.6
4LLS	A	100	holo	4.07	0.81	520.4
1RQI	A	100	holo	4.13	0.66	502.8
3OAC	D	100	holo	4.16	0.75	524.7
2FTZ	A	100	holo	4.21	0.61	494.7
4LFG	A	100	holo	3.95	0.72	509.3
3PDE	C	100	holo	4	0.74	491.2
4FP4	A	100	holo	4.37	0.45	562
2J1P	A	100	holo	4.11	0.74	508.7
3P41	A	100	holo	3.87	0.53	475.6
1RQJ	A	100	holo	4.15	0.64	506.6
3QQV	A	100	holo	4.03	0.65	511.2
3AQC	D	100	holo	4.36	0.56	549.3
5ZE6	A	100	holo	4.26	0.6	522.7
4P0V	A	99.96	holo	4.02	0.84	509.7
6B04	A	99.96	holo	4.2	0.53	498.9
6B04	B	99.96	holo	4.19	0.61	500.8
2E8V	B	99.95	holo	4.61	0.23	529.5
2Z52	A	99.95	holo	5.11	0.51	465.2
3OYR	B	99.95	holo	3.62	0.79	503.3
5CG5	A	99.95	holo	4.15	0.78	480.1
6KD7	A	99.95	holo	4.41	0.77	460.7
4NUA	A	99.95	holo	3.76	0.81	463.4
6R4V	C	99.95	holo	4.53	0.66	541.8
5ERO	A	99.95	holo	4.18	0.52	560.8
5ERO	B	99.95	holo	3.95	1.06	530.2
1UBY	A	99.95	holo	4.6	0.69	494.4
3AQ0	C	99.94	holo	3.97	0.72	513.9
4JZB	B	99.94	holo	4.24	0.86	508
4JZX	A	99.94	holo	4.23	0.83	509.4
4GP2	A	99.94	holo	3.88	0.59	510
4GP2	B	99.94	holo	4	0.63	508.4
1YHL	A	99.94	holo	4.35	0.68	517.5
3ICK	A	99.94	holo	4.32	0.78	517.4
2EWG	A	99.94	holo	4.45	0.67	521
2OGD	A	99.94	holo	4.32	0.71	513.2
3DYH	B	99.94	holo	4.49	0.69	554.4
3PKO	A	99.94	holo	4.73	0.38	534.1
5HN7	E	99.94	holo	3.86	0.94	462.2
5AEL	A	99.92	holo	4.59	0.6	550.6
5AFX	B	99.92	holo	4.68	0.6	551.9

5AHU	D	99.82	holo	4.95	0.57	514.4
